# Cross-Protective Efficacy of Outer Membrane Vesicles (OMVs) Derived from *Salmonella enterica* Serovar Typhimurium Against *Salmonella enterica* Serovars Colonization in SPF Chicken

**DOI:** 10.3390/biology15010011

**Published:** 2025-12-19

**Authors:** Ke Shang, Yu-Ri Choi, Ji-Eun Son, Gyeong-Jun Kim, Jun-Feng Zhang, Ki-Woong Kim, Hyung-Kwan Jang, Bai Wei, Min Kang

**Affiliations:** 1 Luoyang Key Laboratory of Live Carrier Biomaterial and Animal Disease Prevention and Control, College of Animal Science and Technology, Henan University of Science and Technology, Luoyang 471003, China; 2 Department of Avian Diseases, College of Veterinary Medicine and Center for Avian Disease, Jeonbuk National University, Iksan 54596, Republic of Korea; 3 College of Medical Technology and Engineering, Henan University of Science and Technology, Luoyang 471003, China; 4 Bio Disease Control (BIOD) Co., Ltd., Iksan 54596, Republic of Korea

**Keywords:** *Salmonella enterica*, outer membrane vesicles, multidrug resistance, cross-protection, vaccine

## Abstract

Poultry serve as natural reservoirs for *Salmonella* spp., and the consumption of *Salmonella*-contaminated poultry products remains a leading cause of infection in both developed and developing countries. The irrational use of antimicrobials in the poultry industry has contributed to the global emergence of multidrug-resistant (MDR) *Salmonella* strains. This study aimed to evaluate whether *Salmonella enterica* serovar Typhimurium (*S. typhimurium*) outer membrane vesicles (OMVs) could serve as a broad-spectrum immunogen, providing protection against the most prevalent MDR *Salmonella* serovars. The immunogenicity and protective efficacy of *S. typhimurium* OMVs were assessed in both specific-pathogen-free (SPF) mice and chickens challenged with *S. typhimurium* infection. In addition, the cross-protective efficacy of OMVs against infections with heterologous serovars, specifically *S. montevideo* and *S. albany,* was also evaluated in chickens. Our results demonstrated that *S. typhimurium* OMVs elicited a robust humoral immune response and conferred significant immune protection in both mice and chickens. Following challenges with MDR *S. montevideo* and *S. albany* strains, immunized birds exhibited reduced levels of fecal shedding and liver invasion. Furthermore, in vitro studies revealed that *S. typhimurium* OMVs also possess cross-immunogenicity against MDR S. Enteritidis and *S. gallinarum*. In conclusion, *S. typhimurium* OMVs provide cross-protection against heterologous MDR *Salmonella* infections both in vitro and in vivo. Our study suggests that OMVs derived from *S. typhimurium* strains hold promise as novel subunit vaccine candidates for preventing MDR heterologous serovar infections in animals, offering a potential solution to the growing challenge of antimicrobial resistance in the poultry industry.

## Simple Summary

*Salmonella* infections from contaminated poultry are a major global health concern, and overuse of antibiotics in farming has led to dangerous drug-resistant strains. In this study, we investigated whether tiny particles called outer membrane vesicles (OMVs), which are naturally released by *Salmonella* bacteria, could be used as a vaccine to protect against multiple antibiotic-resistant *Salmonella* types. We tested these OMVs in mice and chickens, including against different *Salmonella* strains that infect both animals and humans. Our results showed that OMVs from one *Salmonella* strain could trigger strong immune responses and reduce infections not only from the same strain but also from other drug-resistant strains. Vaccinated chickens had fewer bacteria in their waste and livers, indicating better protection. These findings suggest that OMVs could be a promising new type of vaccine to help control Salmonella in poultry, reducing the need for antibiotics and lowering the risk of foodborne infections in people. This approach may help combat the growing problem of antibiotic resistance while improving food safety worldwide.

## 1. Introduction

Non-typhoid *Salmonella* is one of the main causes of foodborne diseases worldwide, and poultry and their products are the main vectors of transmission, posing a significant public health burden. Faced with the increasingly severe crisis of antimicrobial resistance (AMR), the global public health and veterinary fields are actively seeking alternative strategies to antibiotics (such as vaccines, bacteriophages, etc.) to control pathogens at the source and reduce the use of antibiotics in the poultry industry [1,2,3]. Therefore, multidrug-resistant (MDR) *Salmonella* infections are a concern for the global poultry industry and a threat to the health and safety of human populations worldwide. With the continuous development of anti-substituting and anti-substituting activities in China, the role of vaccines in the prevention and control of *Salmonella* disease has become more and more important. At present, the vaccines used for *Salmonella* prevention and control mainly include live attenuated vaccines and inactivated seedlings, and many subunit vaccines are in the research stage. However, existing commercial vaccines have significant limitations, particularly their inability to provide broad-spectrum protection against diverse serotypes.

Poultry infections are caused by multiple *Salmonella* serotypes, necessitating vaccines that can offer cross-protection. In Korea, a wide diversity of *Salmonella* serotypes, including *S. typhimurium*, *S*. *enteritidis* [4], *S*. *montevideo*, and *S. albany* [5,6], have been isolated from the poultry supply chain. According to recent surveillance reports from the Animal and Plant Quarantine Agency (APQA) in South Korea, the prevalence of two serotypes of *S. montevideo* and *S*. *albany*, in chickens in 2024 was 3.2% (24/749) and 2.3% (17/749), respectively, ranking fourth and sixth in the top 10 [7]. Of particular concern are the emerging multidrug-resistant (MDR) strains of *S. montevideo* and *S. albany*, which are rapidly spreading and pose a significant threat due to the lack of availability of effective vaccines [8]. Existing commercial vaccines primarily target *S*. *enteritidis*, *S. typhimurium*, and *S*. *gallinarum* and offer limited cross-protection against these emerging heterologous serotypes [9,10,11].

Given these challenges, there is a critical need to develop vaccines that can protect against a broader range of *Salmonella* serotypes. Bacterial outer membrane vesicles (OMVs) have emerged as promising vaccine platforms for cross-protection against other serotypes. Bacterial OMVs, which are nanosized (20–250 nm) spherical phospholipid bilayer structures, are naturally released by almost all Gram-negative bacteria and some Gram-positive bacteria [12]. Crucially, OMVs naturally carry multiple conserved antigenic components on their surface, such as OMPs and the LPS core structure. As these antigens share high homology among different *Salmonella* serotypes, OMVs are theoretically capable of inducing robust cross-reactive immune responses [13]. OMVs play various roles, including long-distance signaling, toxin transfer, biofilm formation, defense against competing microbes, antimicrobial response, and envelope stress relaxation [14,15]. OMVs encapsulate materials from their parent bacteria, such as outer membrane proteins (OMPs), periplasmic proteins, phospholipids, lipopolysaccharides (LPSs), DNA, and RNA [16]. Furthermore, OMVs have several advantages including strong immunogenicity, intrinsic adjuvant effects, and safety due to their non-replicating nature [12]. OMV-based vaccines have shown protective effects against various pathogens including *Neisseria meningitidis* [17], avian pathogenic *Escherichia coli* (APEC) O78 [12], *Pseudomonas aeruginosa* [18], *Vibrio cholerae* [19], *Gallibacterium anatis* [20], *Acinetobacter baumannii* [21], *S. typhimurium* [22], and *S*. *enteritidis* [23].

OMVs derived from *S. typhimurium*, which was one of the most prevalence of *Salmonella* spp., have been shown to elicit strong immune responses and provide cross-protection in mouse models [24,25]. However, their effectiveness in poultry, particularly against emerging MDR serotypes such as *S. montevideo* and *S. albany*, has not been extensively studied. In this study, we aimed to evaluate the immunogenicity and protective efficacy of *S. typhimurium* OMVs in specific pathogen-free (SPF) chickens. Our goal was to assess their potential to provide cross-protection against MDR *S. montevideo* and *S. albany* infections, thereby addressing a critical need in the poultry industry for broader and more effective *Salmonella* vaccines.

## 2. Materials and Methods

### 2.1. Statement of Ethics

All experimental and animal management procedures were undertaken in accordance with the requirements of the Animal Care and Ethics Committee of Jeonbuk National University. The animal facility at JBNU is fully accredited by the National Association of Laboratory Animal Care (approval number: CBNU-2020-055).

### 2.2. Chickens and Bacterial Strains

Five-week-old BALB/c mice (Samtako, Daejeon, Korea) and one-week-old SPF White Leghorn chickens (SPAFAS Poultry Company, Jinan, China) were evaluated to confirm the absence of *Salmonella* spp. infection by bacteriological examination and for any clinical signs of enteric disease. The experimental groups were reared in separate positive-pressure isolators in an environmentally controlled facility, and food and water were provided ad libitum. The experiment was conducted under controlled conditions with a temperature of 25 ± 0.3 °C, a relative humidity of 60%, and a 12-h photoperiod [26].

Detailed information of the bacterial strains used in the present study is shown in Table 1. The *S. typhimurium* ATCC 14028 strain was used for OMV vaccine preparation and as the challenge strain. *S. montevideo* A16-CF-111-L-1 and *S. albany* A16-CF-360-1S [27,28] were used as the chicken challenge strains. In addition, strains of *S*. *enteritidis* ATCC 13076, A18-KCI-DEO-1-2S [5], *S*. *gallinarum* 287/91 (NCTC 13346), A17-DW-005 [29], and *E. coli* ATCC 25922 were selected as antigens to detect cross-protective efficacy in vitro.

### 2.3. Isolation and Purification of OMVs from Salmonella Culture Supernatants

**Figure 1:**
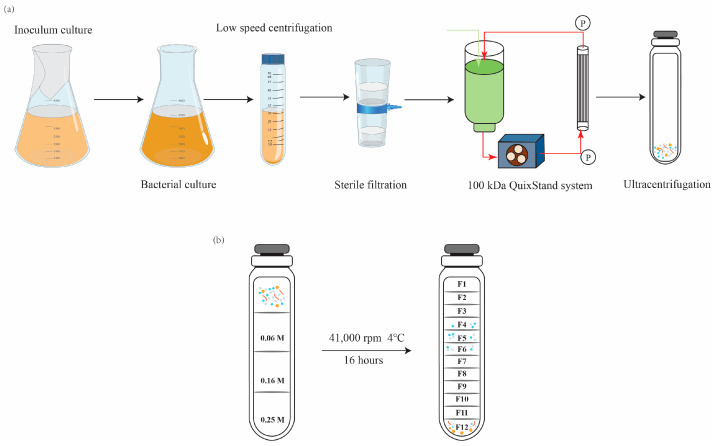
Schematic showing the experimental workflow for (**a**) outer-membrane vesicles (OMVs) extraction and (**b**) purification using a density gradient of sucrose solution.

The production of OMVs is influenced by various factors including growth conditions, stress factors, and growth phases of bacterial cultures. The amount of OMV production can vary in response to these factors. During the late log phase, cells are known to produce a large quantity of OMVs. Therefore, OMVs were isolated from late-log-phase bacterial culture supernatants, as described by Choi et al., with few modifications, as represented graphically in Figure 1a [30]. Briefly, *Salmonella* cells cultured in LB broth (500 mL or 1 L) were centrifuged at 10,000× *g* for 5 min. The cell-free supernatant was then filtered through a polyvinylidene difluoride (PVDF) filter (0.45 μm pore size, Millipore, Burlington, MA, USA) to remove the remaining bacteria. The filtrate was concentrated by ultrafiltration in a stirred diffusion cell (Thermo Fisher Scientific, Waltham, MA, USA) using ultrafiltration disks with a molecular weight cutoff of 100 kDa (MilliporeSigma, Burlington, MA, USA). The retentate was filtered through a 0.45-μm-pore-size PVDF membrane to filter the remaining bacteria, and the filtrate was subjected to ultracentrifugation at 41,000 rpm for 3 h at 4 °C using an SW 41Ti rotor (Optima XE-90, Beckman Coulter, Brea, CA, USA). The supernatant was carefully removed, and the pellet containing vesicles was suspended in 800 μL of Dulbecco’s phosphate-buffered saline. Cell-free preparation of the vesicle fractions was examined by spreading a portion of the vesicles onto LB plates. The OMVs with protein concentrations >1 mg/mL were purified using density gradient of sucrose solution. In brief, the following concentrations of sucrose solution were sequentially added to the tube: 4 mL (0.25 M), 4 mL (0.16 M), and 4 mL (0.06 M), as shown in Figure 1b. Finally, 500 µL of isolated OMVs and normal saline (to fill the tube) was loaded on top of the gradient, and the tubes were subjected to ultracentrifugation at 41,000 rpm for 16 h at 4 °C. The different gradient layers were then collected, and each layer was ultracentrifuged at 41,000 rpm for 4 h at 4 °C; all pellets were resuspended in saline.

### 2.4. Characterization of OMVs

The protein concentration in the extracted OMVs was measured using a BCA Protein Assay kit (Thermo Fisher Scientific). Nanoparticle tracking analysis (NTA) was conducted using a ZetaView PMX 110 instrument (Particle Metrix GmbH, Inning am Ammersee, Germany) according to the manufacturer’s instructions. The instrument was calibrated against a known concentration of PS100 nanoparticles with a diameter of 100 nm (Applied Microspheres B. V., Utrecht, The Netherlands). Nanostandards and OMV samples were suspended in particle-free phosphate-buffered saline (PBS) (Sigma-Aldrich, St. Louis, MO, USA) and diluted appropriately before analysis. Each sample was counted and sized across 2 cycles of 11 frames per cycle with a flow cell sensitivity of 80%. The OMV samples were placed onto a 200-mesh copper grid, negatively stained with 4% uranyl acetate, and visualized using an HT7700 transmission electron microscope (TEM) to determine the size of the OMVs (Hitachi, Tokyo, Japan) [31].

### 2.5. Immunization Protocol and Challenge

**Figure 2:**
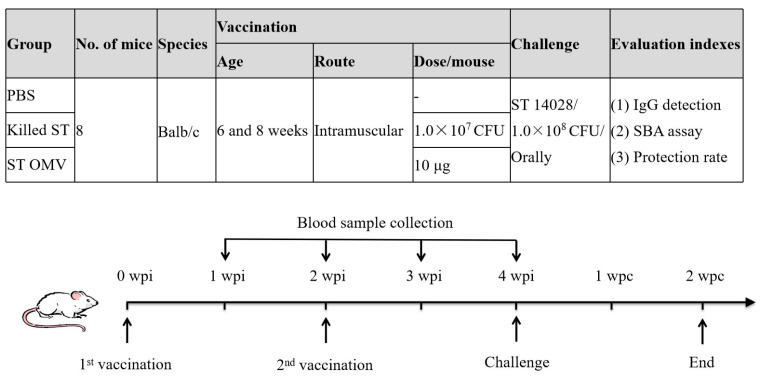
Timeline of immunization and challenge in a mice experiment to evaluate the protective efficacy of OMVs from *S. typhimurium*. Three groups of mice were separately immunized with OMVs (10 μg of OMVs based on protein content in 100 μL of PBS per animal), killed *S. typhimurium* or PBS (as a control) on day 0, boosted at week 2, and challenged at 4 weeks post the first immunization. Each group consisted of 6 mice. Blood samples were collected weekly. Before immunization, all mice blood was drawn for testing whether the blood was contaminated by bacteria. PBS vaccinated mice served as a negative control group.

Eight mice of approximately equal weights were divided into groups for intramuscular immunization (10 μg of OMVs based on protein content in 100 μL of PBS per animal), which were performed twice with Freund’s adjuvant Incomplete use (Sigma-Aldrich, St. Louis, MO, USA) (Figure 2). The vaccine control group was immunized with a formalin-treated *S. typhimurium* killed vaccine (10^7^ CFU in 100 μL of PBS per animal), which were also performed twice with Freund’s adjuvant Incomplete use also. The negative control group was administered the equivalent volume of PBS. To determine the systemic immunity induced by OMVs, blood samples were collected from the mice via orbital sinus puncture at one-week intervals after the first immunization. The blood serum was centrifuged and stored at −80 °C for future use. To calculate the rate of protection, two weeks after the booster immunization, the mice were challenged with a lethal dose of *S. typhimurium* ATCC 14028 (1.0 × 10^8^ CFU, determined by continuously diluting and then counting on the LB agar plate). Mice were monitored for their weights as well as signs of illness, obvious discomfort, distress, or pain. Given the humane endpoint and euthanasia standards, mice that exhibited weight loss of greater than 30% of their starting weights were euthanized via carbon dioxide.

**Figure 3:**
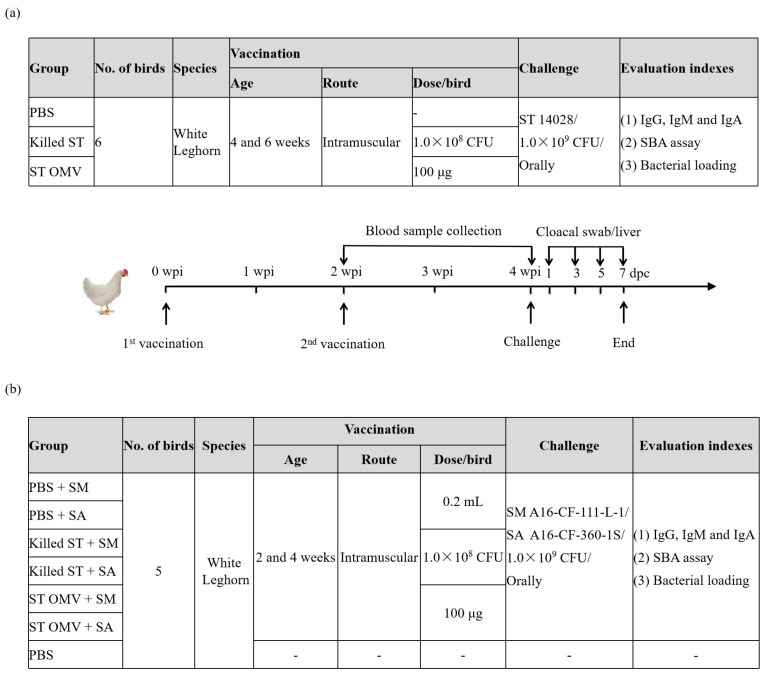
Timeline of immunization and challenge in a chicken experiment to evaluate the cross-protective efficacy of OMVs from *S. typhimurium*. To evaluate the protective efficacy of *S. typhimurium* OMVs in chickens, 2-week-old SPF White Leghorn chickens were immunized intramuscularly (100 μg of OMVs in 200 μL of PBS per bird) at 2-week intervals as potential immune-protective administration, and 200 μL of PBS was used as a negative control, each group consisted of six birds (**a**). To evaluate the cross-protective efficacy of *S. typhimurium* OMVs in chickens, 4-week-old SPF White Leghorn chickens were immunized intramuscularly (100 μg of OMVs in 200 μL of PBS per bird) at 2-week intervals as potential immune-protective administration, and 200 μL of PBS was used as a negative control, each group consisted of five birds (**b**). Blood samples were collected weekly. Before immunization, all bird blood was drawn for testing whether the blood was contaminated by bacteria. PBS vaccinated birds served as a negative control group.

To evaluate the protective efficacy of *S. typhimurium* OMVs in chickens, 2-week-old SPF White Leghorn chickens were immunized intramuscularly (100 μg of OMVs in 200 μL of PBS per bird) at 2-week intervals as potential immune-protective administration, and 200 μL of PBS was used as a negative control (Figure 3a). The day of the first immunization was marked as day 0, and the booster day was marked as week 2. Approximately 0.5–1 mL of blood was aseptically drawn from the chickens’ brachial veins weekly after the first immunization. Then, the collected blood was incubated at room temperature for 1 h, the blood clot was removed with sterile toothpicks, and the sample was centrifuged at 1000× *g* for 15 min. The supernatants were gently removed, and the prepared serum samples were stored at −80 °C. To calculate the rate of protection, two weeks after the booster immunization, the birds were challenged with *S. typhimurium* ATCC 14028 (1.0 × 10^9^ CFU, determined by continuously diluting and then counting on the LB agar plate). No mortality occurred because this strain did not cause the death of the chickens in our studies. Therefore, fecal shedding and bacterial loading in the liver of the challenge strain were monitored to evaluate the protective efficacy against *S. typhimurium*. For bacterial enumeration, feces collected by a cloacal swab and/or a liver tissue (1 g) sample were suspended in BPW buffer, and 10-fold serially diluted suspensions were plated on Brilliance *Salmonella* agar (Oxoid Ltd., Basingstoke, UK) supplemented with 1 μg/mL novobiocin and 50 μg/mL nalidixic acid (Oxoid Ltd., Basingstoke, UK). The colonies were counted after 24–48 h, and 3–5 colonies were randomly selected for agglutination with O:4 (B) serum (Oxoid Ltd., Basingstoke, UK).

To evaluate the cross-protective efficacy of *S. typhimurium* OMVs in chickens, 4-week-old SPF White Leghorn chickens were immunized intramuscularly (100 μg of OMVs in 200 μL of PBS per bird) at 2-week intervals as potential immune-protective administration, and 200 μL of PBS was used as a negative control (Figure 3b). To calculate the rate of protection, two weeks after the booster immunization, the birds were challenged with *S. montevideo* A16-CF-109-1S-1, and *S. albany* A16-CF-360-1S (1.0 × 10^9^ CFU). The experimental implementation plan is the same as above (Figure 3a).

### 2.6. Enzyme-Linked Immunosorbent Assay (ELISA)

The titers of anti-*S. typhimurium* IgG (IgM, IgA) in serum samples were measured by ELISA, as described previously, with some modifications [22]. An ultrasonicated whole cell extract of the bacteria was used for coating antigen. Briefly, 96-well plates were coated, washed and blocked as follows: the plates were coated overnight at 4 °C with 100 ng of *S. typhimurium* ultrasonic antigen in 100 μL of coating buffer (0.016 M Na_2_CO_3_, 0.034 M NaHCO_3_ [pH 9.6]), the coating solution was removed, and they were washed twice with 350 μL of washing buffer (PBS + 0.05% Tween 20) and then blocked for 2 h at 37 °C with 200 μL of blocking buffer (washing buffer + 2% bovine serum albumin [BSA]). A total of 100 μL of serum samples was diluted in dilution buffer (PBS + 2% BSA) at 1:400 and incubated in wells for 1 h at 37 °C with 100 μL of dilution buffer as a negative control. Then, 100 μL of 1:8000 horseradish peroxidase (HRP)-conjugated rabbit anti-mouse-IgG gamma and HRP-conjugated goat anti-chicken IgG (H+L) (KPL, Milford, MA, USA) or 1:10,000 HRP-conjugated goat anti-chicken IgA antibody and HRP-conjugated goat anti-chicken IgM antibody (Bethyl Laboratories, Tuas, Singapore) in dilution buffer was added to the wells and further incubated for 1 h at 37 °C. Subsequently, 100 μL of TMB substrate was transferred to the wells and reacted for 1 h at room temperature. Then, 50 μL of stop solution (2.25 M H_2_SO_4_) was added to terminate the reaction. The OD_450_ was measured immediately in an ELISA plate reader (PerkinElmer Inc., Shelton, CT, USA). All samples were independently run in triplicate, and the logarithmic antibody titers were calculated for further analysis.

### 2.7. Serum Bactericidal Activity (SBA) Assay

**Figure 4:**
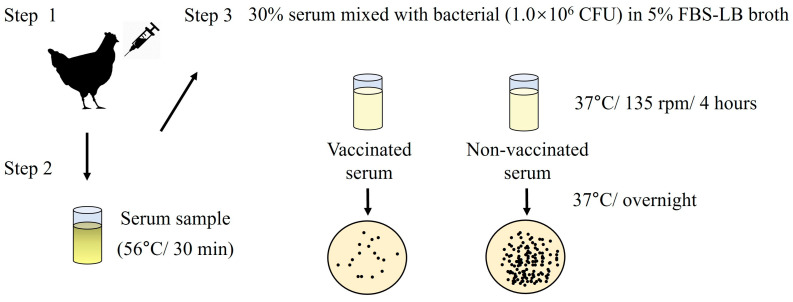
Schematic illustrating serum bactericidal activity (SBA) assay.

A modified serum SBA assay was performed to evaluate the bactericidal ability of antibodies in serum samples in vitro (Figure 4) [32]. An overnight culture of selected strains (*S. typhimurium* ATCC 14028; *S*. *gallinarum* NCTC 13346 and A17-DW-005; *S*. *enteritidis* ATCC 13076 and A18-KCI-DEO-1-2S; *E. coli* ATCC 25922) to the logarithmic phase (OD_600_ = 0.4–0.6). The bacteria were adjusted to 1.0 × 10^6^ CFU and incubated with 30% heat-inactivated (56 °C for 30 min) serum in LB broth containing 5% fetal bovine serum (FBS) (Sigma-Aldrich, St. Louis, MO, USA) in a 24-well plate (135 rpm at 37 °C). Bacteria incubated in 5% FBS-LB alone were used as negative controls. Four hours after incubation, 10-fold serial dilutions were plated on LB agar and incubated for 12–24 h at 37 °C. The bacterial counts were reported as CFU/mL. Each experimental group was assayed in triplicate, and two independent experiments were performed.

### 2.8. Statistical Analysis

The data were expressed as the mean ± standard deviation (SD). The differences between the immunized groups and the PBS control group were compared using Student’s *t*-test and one-way ANOVA. A *p*-value of ≥0.05 was considered statistically non-significant (ns). *p* values < 0.05 were considered significant and are represented as ‘*’; *p* values < 0.01 were considered significant and are represented as ‘**’. Data has passed the normality test (Shapiro–Wilk test) and homogeneity of variance test to meet the assumptions of parameter testing.

## 3. Results

### 3.1. Isolation and Characterization of S. typhimurium OMVs

**Figure 5:**
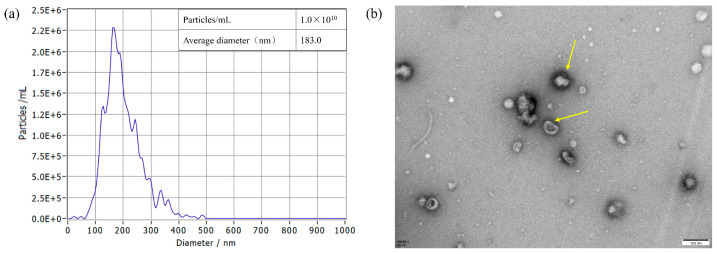
OMVs isolated from *S. typhimurium* strain were diluted 400-fold and measured using a ZetaView PMX 110 instrument (Particle Metrix GmbH, Starnberg, Germany). All data represent the mean of triplicate experiments ± standard error (**a**). Determination of the size of OMVs from *S. typhimurium* strains using TEM analysis (**b**). TEM image of the OMVs, with the yellow arrow indicating the OMVs. Scale bar = 100 nm.

The protein concentration results showed that the protein concentration of *S. typhimurium* OMVs was 2.0 mg/mL. The OMVs had an average diameter of 183.0 nm, and their size ranged between 50 and 350 nm in diameter when measured using NTA (Figure 5a). TEM images revealed that *S. typhimurium*-secreted OMVs were spherical in nature and contained electron-dense structures with various shapes and sizes (Figure 5b).

### 3.2. IgG Immune Responses Induced by S. typhimurium OMVs in Mice

**Figure 6:**
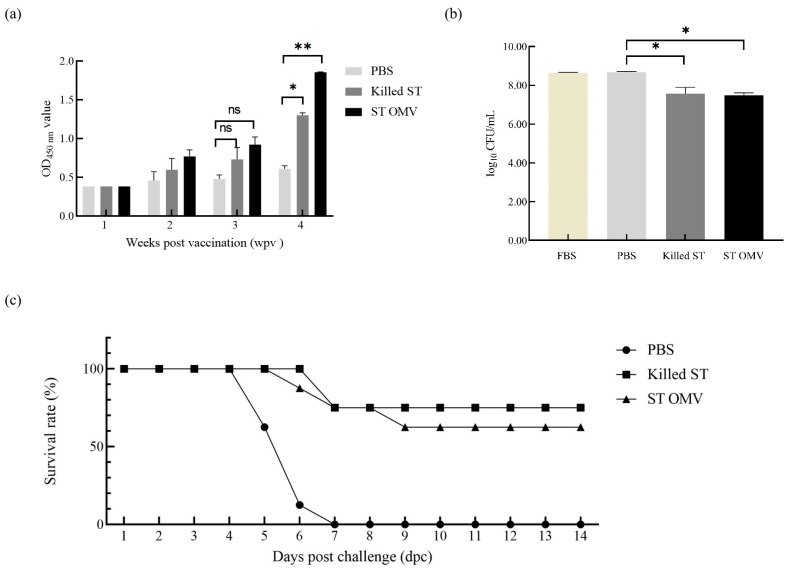
Immunoprotected effect in mice. (**a**) IgG immune responses in mice. The titers of anti *S. typhimurium* IgG in sera from mice immunized with killed *S. typhimurium*, and OMVs. (**b**) Bactericidal ability of immunized mice serum. Bacterial survival after incubation with OMV-, killed *S. typhimurium*- and PBS-immunized serum samples at 37 °C for 4 h. (**c**) Survival of immunized mice after challenge with wild-type *S. typhimurium*. Immunization with OMVs derived from wild-type *S. typhimurium* provided 62.5% protection against a lethal-dose (10^8^ CFU) *S. typhimurium* strain challenge in mice. All the mice were monitored daily for 2 weeks after challenge for morbidity and mortality. Each experiment was performed with three technical replicates. A student *t*-test and one-way ANOVA were performed for statistical analysis; error bar type was mean ± standard error. A *p*-value of ≥ 0.05 was considered statistically non-significant (ns). *p* < 0.05 refers to statistically significant, annotated as *, *p* values < 0.01 were considered significant and are represented as ‘**’.

To determine the immunogenicity of OMVs from *S. typhimurium*, the levels of anti-*S. typhimurium* IgG in serum samples were measured by ELISA. IgG developed two weeks after the first vaccination in both the killed *S. typhimurium* and OMV groups. The OMV and *S. typhimurium* killed vaccine groups showed similar levels, and the antibody levels increased over time (Figure 6a). Analysis of variance revealed significantly higher anti-*S. typhimurium* IgG titers in both the killed *S. typhimurium* and OMV groups compared to the PBS control at four weeks post-immunization (*p* = 0.013).

### 3.3. SBA Induced by S. typhimurium in Mice

To test the bactericidal activity of serum in the OMV-immunized mice, a modified SBA assay was utilized. The serum samples derived from PBS-, killed *S. typhimurium*-, and OMV-immunized mice at 4 weeks after the first immunization were incubated with *S. typhimurium* bacteria at 37 °C for 4 h. Then, the bacterial survival after different treatments was statistically analyzed (Figure 6b). Analysis of variance revealed that, compared to the PBS control sera, sera from both the killed *S. typhimurium* and OMV groups displayed significant bactericidal activity (*p* = 0.026), whereas no such activity was detected in the PBS group (*p* = 0.532).

### 3.4. Protection Against Virulent S. typhimurium Challenge in Mice

To evaluate the protective efficacy of *S. typhimurium* OMVs, immunized mice in different groups were challenged via the oral route with 1.0 × 10^8^ CFU (LD_100_) of *S. typhimurium* two weeks after booster immunization. Immunization with OMVs derived from *S. typhimurium* provided mice with 62.5% (5 out of 8 survived) protection against a challenge with *S. typhimurium*, while mice immunized with killed *S. typhimurium* had 75% survival (6 out of 8 survived), and all the mice in the PBS control group succumbed to infection with the *S. typhimurium* strain within 7 days (Figure 6c).

### 3.5. IgG, IgM, and IgA Immune Responses Induced by S. typhimurium OMVs in Chicken

**Figure 7:**
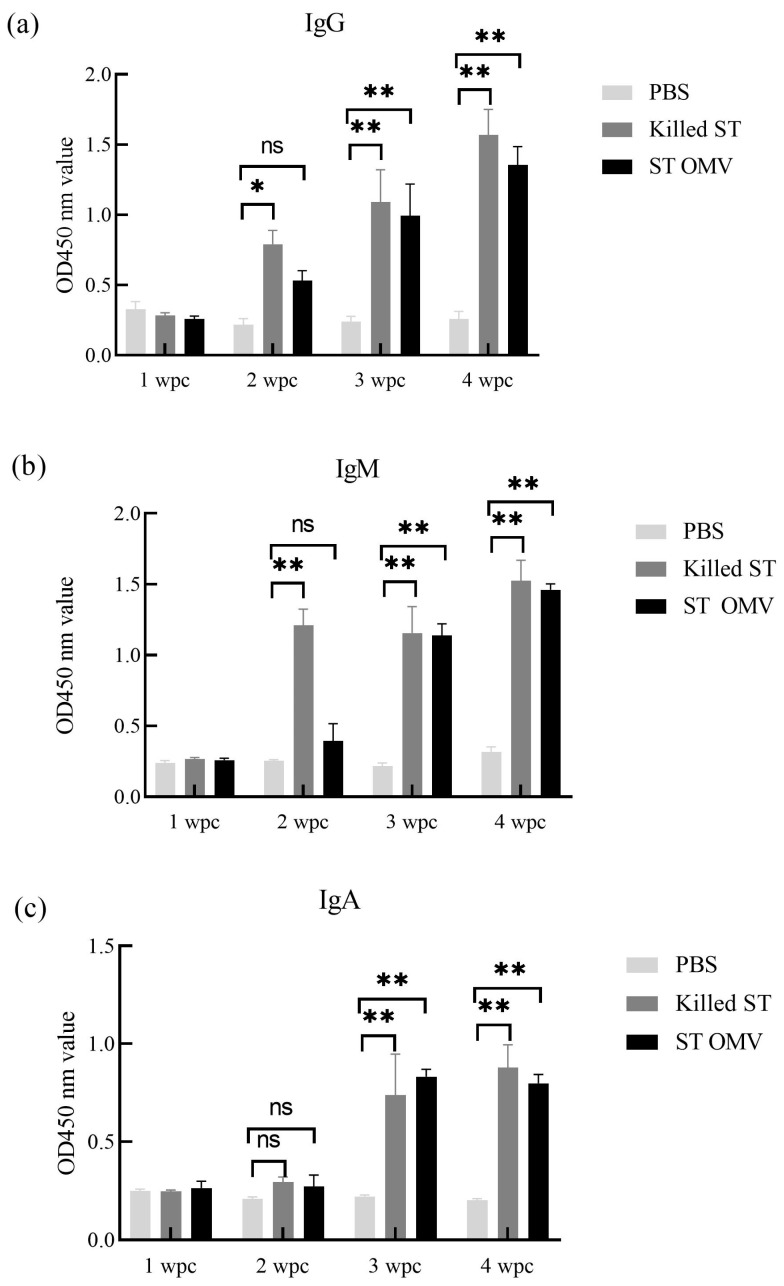
IgG, IgM, and IgA immune responses in chicken. The titers of anti *S. typhimurium* IgG (**a**), IgM (**b**), and IgA (**c**) in sera from mice immunized with killed *S. typhimurium*, and OMVs. Each experiment was performed with three technical replicates. A student *t*-test and one-way ANOVA were performed for statistical analysis; error bar type was mean ± standard error. A *p*-value of ≥ 0.05 was considered statistically non-significant (ns). *p* < 0.05 refers to statistically significant, annotated as *, *p* values < 0.01 were considered significant and are represented as ‘**’.

To determine the immunogenicity of OMVs from *S. typhimurium*, the levels of anti-*S. typhimurium* IgG, IgM, and IgA in serum samples were measured by ELISA. The OMV and *S. typhimurium* killed vaccine groups showed similar levels, and the antibody levels of IgG, IgM, and IgA increased time dependence. At three and four weeks after the first immunization, both the killed *S. typhimurium* and OMV groups had significantly higher anti-*S. typhimurium* IgG (Figure 7a) production than the PBS control groups (*p* < 0.05). Two weeks after the first immunization, the killed *S. typhimurium* groups had significantly higher anti-*S. typhimurium* IgM (Figure 7b) production than the PBS control groups (*p* < 0.05). At three and four weeks after the first immunization, both the killed *S. typhimurium* and OMV groups had significantly higher anti-*S. typhimurium* IgM production than the PBS control groups (*p* < 0.05). Finally, three and four weeks after the first immunization, both the killed *S. typhimurium* and OMV groups had significantly higher anti-*S. typhimurium* IgA (Figure 7c) production than the PBS control groups (*p* < 0.05).

### 3.6. Reduction in MDR S. montevideo and S. albany in the Cloacae and Livers of Challenged Chickens After S. typhimurium OMV Vaccination

Immunized chickens in the different groups were challenged via the oral route with 1.0 × 10^9^ CFU of *S. typhimurium*, *S*. Montevideo, and *S. albany* four weeks after immunization to evaluate the protective efficacy of *S. typhimurium* OMVs. In the first animal trial, no clinical symptoms or death were found in the chickens during the whole experiment. In the PBS immunization group, the positive rate in cloacal swab samples was 100% (6/6) at 1, 3, 5, and 7 days post-challenge (Table 2). The positive rates of cloacal swab samples in the inactivated *S. typhimurium* vaccine group and the OMV immunized group were similar, and both were lower than that in the PBS immunized group. At seven days post-challenge, the ratio of positive/total cloacal swabs was lower in inactivated *S. typhimurium* (2/6) and OMV-vaccinated chickens (1/6) than in PBS-vaccinated chickens (6/6). At seven days post-challenge, the *S. typhimurium* log_10_ CFU/g of cloacal swabs was lower in vaccinated chickens (0.00 ± 0.00) than in PBS-vaccinated chickens (2.50 ± 0.64). Similarly, the ratio of positive/total liver samples was lower in chickens vaccinated with inactivated *S. typhimurium* (2/6) and OMV-vaccinated chickens (3/6) than in PBS-vaccinated chickens (5/6); the *S. typhimurium* log10 CFU/g of liver samples was lower in vaccinated chickens (0.00 ± 0.00) than in PBS-vaccinated chickens (1.46 ± 1.46) at seven days post-challenge.

In the second animal trial, chickens were vaccinated (at 2 weeks old) and booster vaccinated (at 4 weeks old) with OMVs derived from *S. typhimurium* SL14028 (*n* = 5); age-matched chickens were PBS-vaccinated with PBS (*n* = 5). At 6 weeks of age, the chickens were separately challenged with 1 × 10^9^ CFU of MDR *S. montevideo* and *S. albany*. For *S*. Montevideo-challenged chickens, the positive rate of cloacal swab samples in the OMV-immunized group were lower than that in the PBS-immunized group (Table 3). Five days post-challenge, the ratio of positive/total cloacal swabs in OMV-vaccinated chickens (2/5) was lower than in PBS-vaccinated chickens (3/5). Seven days post-challenge, the ratio of positive/total cloacal swabs was lower in OMV-vaccinated chicken (0/5) than in PBS-vaccinated chickens (1/5). Seven days post-challenge, the *S. typhimurium* log10 CFU/g of cloacal swabs was lower in OMV-vaccinated chickens (0.00 ± 0.00) than in PBS-vaccinated chickens (1.36 ± 1.17). Similarly, the ratio of positive/total liver samples was lower in OMV-vaccinated chickens (0/5) than in PBS-vaccinated chickens (2/5). In addition, the *S. typhimurium* log_10_ CFU/g of liver samples were lower in OMV-vaccinated chickens (0.00 ± 0.00) than in PBS-vaccinated chickens (0.68 ± 0.83) seven days post-challenge. For *S. albany* -challenged chickens, the positive rates of cloacal swab samples in the OMV-immunized group was lower than that in the PBS-immunized group (Table 3). Five days post-challenge, the ratio of positive/total cloacal swabs was lower in OMV-vaccinated chickens (3/5) than in PBS-vaccinated chickens (5/5). Seven days post-challenge, the ratio positive/total cloacal swabs was lower in OMV-vaccinated chickens (2/5) than in PBS-vaccinated chickens (5/5). Seven days post-challenge, the *S. typhimurium* log_10_ CFU/g of cloacal swabs was lower in OMV-vaccinated chickens (1.20 ± 1.49) than in PBS-vaccinated chickens (2.13 ± 0.50). Similarly, the ratio of positive/total sliver sample was lower in OMV-vaccinated chickens (0/5) than in PBS-vaccinated chickens (1/5). The *S. typhimurium* log_10_ CFU/g of liver samples were lower in OMV-vaccinated chickens (0.00 ± 0.00) than in PBS-vaccinated chickens (0.34 ± 0.68) at seven days post-challenge.

### 3.7. Bactericidal Ability Against Multiple Strains in Vitro

**Figure 8:**
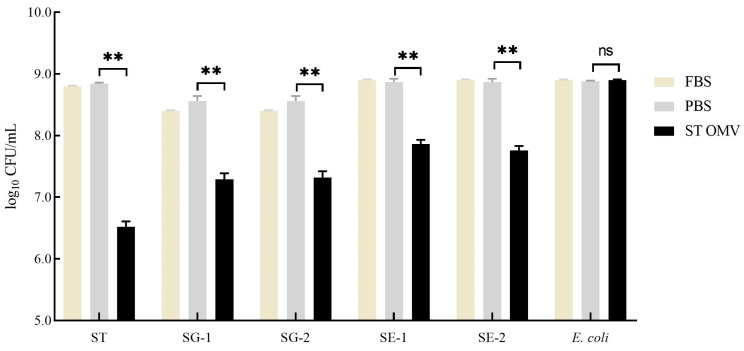
Bactericidal ability of immunized chicken serum. Bacterial (*S. typhimurium*, *Gallinarum*, *Enteritidis*, and *E. coli*) survival after incubation with OMV- and PBS-immunized serum samples at 37 °C for 4 h. Each experiment was performed with three technical replicates. A student t-test and one-way ANOVA were performed for statistical analysis; error bar type was mean ± standard error. A *p*-value of ≥ 0.05 was considered statistically non-significant (ns). *p* values < 0.01 were considered significant and are represented as ‘**’.

To test the bactericidal activity of serum in the OMV-immunized chicken, a modified SBA assay was utilized. The serum samples derived from PBS- and OMV-immunized chickens at 4 weeks after the first immunization were incubated with bacteria (*S. typhimurium* ATCC 14028; *S. gallinarum* NCTC 13346 [SG-1] and A17-DW-005 [SG-2]; *S*. *enteritidis* ATCC 13076 [SE-1] and A18-KCI-DEO-1-2S [SE-2]; *E. coli* ATCC 25922) at 37 °C for 4 h. Then, the bacterial survival with different treatments was statistically analyzed (Figure 8). The bacterial survival in serum samples from the PBS-immunized group did not significantly change (*p* > 0.05). For the *S. typhimurium* inoculation group, serum samples from OMV immunization group significantly suppressed the growth of bacteria (*p* < 0.05) compared with serum samples from the PBS control group. In the *S*. *gallinarum* inoculation groups, serum samples from OMV immunization groups showed significantly bactericidal activity (*p* < 0.05) compared with serum samples from the PBS control group. Similarly, in the *S*. *enteritidis* inoculation groups, serum samples from OMV immunization groups showed significant bactericidal activity (*p* < 0.05) compared with serum samples from the PBS control group. However, the bacterial counts in serum samples from the PBS-immunized group and immunization group did not significantly change in the *E. coli* inoculation groups (*p* > 0.05).

## 4. Discussion

*Salmonella* spp., which are common foodborne pathogens, pose a significant threat to global public health, and the emergence of MDR *Salmonella* strains has exacerbated this threat. Current *Salmonella* vaccines, while somewhat effective in reducing the incidence of infections, are limited by their lack of cross-serotype protection [13,33,34]. OMV-based vaccines have emerged as a promising new approach to combat bacterial infections due to their high immunogenicity, high safety, and cross-protection potential, which makes them a potent tool in the fight against bacterial pathogens. In this study, we aimed to evaluate, for the first time, the cross-protection offered by *S. typhimurium* OMVs against infections caused by the current prevalent MDR *S. montevideo* and *S. albany* strains from Korea using a chicken model.

In this study, OMVs derived from *S. typhimurium* ATCC 14028 were characterized by an average diameter of 183.0 nm (Figure 5a) and exhibited a spherical morphology with electron-dense structures of varying shapes and sizes (Figure 5b), consistent with previous findings [35]. The structural similarity between OMVs and the bacterial outer membrane is crucial as it enables OMVs to retain a comparable level of immunogenicity to their bacterial parents [12,24]. Furthermore, our findings demonstrate that OMVs derived from *S. typhimurium* effectively stimulated the host immune system, inducing a robust humoral immune response (Figure 6a,b) and providing protective efficacy (Figure 6c) in a murine model [6,36]. We further evaluated the immune protection provided by *S. typhimurium* OMVs using a chicken model. Consistent with the results observed in mice, *S. typhimurium* OMVs effectively stimulated the host immune system in chickens, leading to the production of protective antibodies. These findings reinforce the notion that naturally released OMVs, which share structural similarities with their bacterial parent, possess strong immunogenicity and can elicit a robust adaptive immune response in chickens. Moreover, our results confirm that the immunogenic capabilities of OMVs observed in mice are similarly present in chickens, an area of study for which data have been relatively scarce until now. Furthermore, our experiments demonstrated that OMV vaccination not only significantly reduced the invasion of internal organs but also decreased the fecal shedding of *Salmonella*. Since *S. typhimurium* infection is a systemic disease transmitted via the oral-fecal route, reducing fecal *Salmonella* levels is a critical strategy for controlling the spread of infection. This protective effect is likely attributable to the presence of both antigens and adjuvants, such as LPS and lipoproteins, within the OMVs [37]. These components interact directly with various immune cells—including neutrophils, macrophages, and dendritic cells—upon immunization, enhancing antibody production and T-cell responses, as noted by Kaparakis-Liaskos [38]. Therefore, our results strongly suggest that immunization with *S. typhimurium* OMVs induces a potent adaptive immune response, providing significant protection against *S. typhimurium* infection in both mouse and chicken models. This underscores the potential of OMV-based vaccines as a valuable tool in combating *Salmonella* spp. infections across different hosts.

Compared to traditional therapeutic and prophylactic strategies, OMV-based vaccines offer distinct advantages. Structurally, the spherical nature and nanoscale size of OMVs allow them to exist stably in the host and efficiently enter the lymphatic system, facilitating uptake by antigen-presenting cells (APCs). This addresses the limitations of traditional inactivated vaccines, which are often prone to rapid clearance [39]. Unlike subunit vaccines that require external adjuvants, OMVs possess intrinsic adjuvant properties [40]. Their surface carries pathogen-associated molecular patterns (PAMPs), such as LPS, peptidoglycan, and lipoproteins. These components effectively induce the expression of CD86 and MHC-II on dendritic cells and activate transcription factors like NF-κB. This activation promotes the release of pro-inflammatory cytokines, including TNF-α, IL-1β, IL-6, and IL-12, thereby enhancing the systemic immune response. Furthermore, in comparison to live attenuated vaccines, OMVs present a superior safety profile [41]. As non-replicating particles, they eliminate the risk of reversion to virulence while retaining the antigen in its natural state without the need for chemical inactivation. Finally, the production of *Salmonella* OMV-based vaccines is cost-effective and scalable, as antigens can be biosynthesized directly by engineered bacteria, making them a practical solution for the poultry industry [42].

In addition to their primary protective effects, OMV vaccines have demonstrated the ability to confer cross-protection against different *Salmonella* serotypes. Our experimental results revealed that *S. typhimurium* OMVs provided cross-protection against *S. montevideo* and *S. albany* infections (Table 3). This may be because there are two major OMV antigen types: conserved OMPs and conserved lipid A core moiety. OMPs isolated from the *S. typhimurium* with truncated LPS were capable of inducing cross-protective immunity against *S. typhimurium* and heterologous serovars [35]. In birds immunized with *S. typhimurium* OMVs, there was a notable reduction in bacterial loads not only in internal organs such as the liver but also in fecal samples, indicating a broad protective efficacy. Notably, serovars *S. montevideo* (serogroup C1) and *S. albany* (serogroup C2–C3) belong to different serological groups compared with *S. typhimurium* (serogroup B) based on their antigenic formulas. Our understanding of the specific structural and functional components of OMVs remains incomplete, and it is currently unclear which protein components within the OMVs are primarily responsible for inducing cross-immunity and conferring cross-protection [43]. However, the observed results are consistent with those of previous studies, which have shown that OMVs, particularly those derived from *S. typhimurium*, are capable of promoting cross-reactive antibodies and providing cross-protection against various *Salmonella* serotypes [43,44]. As the challenge of vaccine development often lags behind the emergence of prevalent strains, these findings are particularly significant. In South Korea, *S. montevideo* and *S. albany* have emerged as the dominant circulating strains, with MDR variants spreading throughout the poultry production chain, posing a serious threat to public health. The ability of *S. typhimurium* OMVs to control not only *S. typhimurium* but also *S. montevideo* and *S. albany* provides a promising strategy for using either single- or mixed-serotype OMVs to combat multiple, or even all, *Salmonella* serotypes. This insight marks a crucial step forward in the development of broad-spectrum vaccines that can address the evolving landscape of *Salmonella* infections.

We additionally evaluated the cross-protective potential of *S. typhimurium* OMVs against two significant serotypes: *S*. *gallinarum*, which poses a severe threat to the poultry industry, and *S*. *enteritidis*, which is a major concern for public health [45]. This assessment was conducted using the SBA, a well-established method for evaluating vaccine efficacy. Our results indicate that *S. typhimurium* OMVs indeed possess the potential to combat these two serotypes (Figure 8). These findings are consistent with those of previous studies that found that *S. typhimurium* OMVs provide protection against *S*. *enteritidis* in chickens [23]. Given that *S*. *enteritidis* and *S*. *gallinarum* belong to the same serogroup D, these results further affirm the validity of the protective efficacy observed in our study.

Unexpectedly, however, the SBA results indicated no protective effect against APEC. This outcome contradicts the findings of prior animal studies that reported protective effects of OMVs against APEC [12]. Since similar SBA results have not been previously documented, we cannot conclusively determine whether the current findings reflect an underlying issue with the SBA assay in this context or if other factors are at play. We propose several hypotheses to explain this discrepancy. First, antigenic heterogeneity implies that the specific bacterial strains used in our SBA may differ from those used in earlier APEC studies, potentially leading to different outcomes. In addition, it is important to consider that OMVs can induce not only serum antibodies but also mucosal and cellular immune responses. The SBA assay primarily assesses the bactericidal activity of serum antibodies, which may not fully capture the protective mechanisms conferred by OMVs against APEC in poultry. Second, APEC may possess specific evasion mechanisms; for instance, its capsule can act as a physical barrier masking surface antigen [46]. Moreover, APEC may have evolved enhanced resistance to serum complement-mediated killing [47]. Therefore, while our current findings suggest a lack of efficacy against APEC in the SBA assay, they do not definitively rule out the potential of *S. typhimurium* OMVs to protect against APEC through other immune pathways. Future research will be necessary to further investigate these issues and clarify the full spectrum of immune responses elicited by *S. typhimurium* OMVs, particularly against diverse bacterial pathogens in poultry such as APEC and *Pasteurella multocida*. These efforts will help refine our understanding of OMV-based vaccines and their role in protecting against a broader range of serotypes and pathogens [48].

While our study successfully demonstrated the protective effects of OMVs, it is important to acknowledge that we did not directly assess the cellular immune response following OMV immunization. However, previous research has already shown that OMVs are capable of inducing a strong cellular immune response, which is crucial for effectively clearing *Salmonella* infections [49,50]. This established understanding supports the potential of OMV-based vaccines, particularly in enhancing the ability of the immune system to combat systemic infections. Another limitation of the present study is that the number of experimental animals used is small (5 or 6 chickens per group) in both chicken vaccination and virulence challenge tests, and we will increase the number of chickens in each group in future research and once again verify the accuracy of our experimental results. Our focus on intramuscular immunization in this study was deliberate because it effectively induced specific antibodies and conferred protection against *Salmonella* spp. in both mice and chickens. However, it is worth noting that prior studies have demonstrated the efficacy of alternative immunization routes, such as oral or intranasal administration, in generating robust immune responses [51,52]. These routes are especially beneficial as they stimulate mucosal immunity, which also plays a critical role in defending against intestinal *Salmonella* spp. infections. Given these findings, there is a compelling case for combining oral or intranasal immunization with intramuscular administration to enhance both mucosal and systemic immunity. Such a combined approach could offer a more comprehensive protective effect since an oral-intramuscular prime-boost strategy may optimize antigen presentation and promote the expansion of antigen-specific CD4+ T cells and B cells [53,54]. This could result in a more robust and well-rounded immune response, further enhancing the protective efficacy of the vaccine. By investigating and optimizing these combined immunization strategies, we can potentially develop OMV-based vaccines that offer even broader and more effective protection against *Salmonella* spp. infections across different serotypes and host species.

## 5. Conclusions

In conclusion, our study successfully prepared OMVs from *S. typhimurium* ATCC 14028 strains and thoroughly investigated their immunogenicity in both mouse and chicken models. Our findings demonstrated that OMVs derived from *S. typhimurium* are capable of inducing a robust adaptive immune response and against *S. typhimurium* infection in mice and chickens. Notably, these OMVs provided cross-protection against challenges from field-prevalent MDR *S. montevideo* and *S. albany* strains in chickens, highlighting the broad protective potential of this vaccine approach. These results suggest that *S. typhimurium* OMVs hold promise as a potential multivalent vaccine candidate, particularly effective against these specific heterologous serotypes. However, while these findings are encouraging, further research involving a wider array of serotypes is necessary to fully confirm and establish the broad-spectrum efficacy of this vaccine platform. Future research should focus on optimizing immunization strategies and further elucidating the mechanisms underlying the immune responses induced by OMV vaccines. This could pave the way for the creation of more effective and versatile vaccines, offering enhanced protection against *Salmonella* spp. infections in both poultry and potentially other host species.

## Figures and Tables

**Table 1:** The *Salmonella* enterica strains used in the present study.

No.	Serotype (Serogroup)	Strain	Antimicrobial Resistance	Source/Host	Purpose of Use	Reference
1	*Typhimurium* (B)	14028	Susceptible	NA/Chicken	Challenge	ATCC
2	*Montevideo* (C1)	A16-CF-111-L-1	NAL-NEO-STR-TET	Litter/Chicken	Challenge	[27]
3	*Albany* (C2–C3)	A16-CF-360-1S	NAL-SXT	Feces/Chicken	Challenge	[28]
4	*Enteritidis* (D)	13076	Susceptible	NA/NA	SBA assay	ATCC
5	*Enteritidis* (D)	A18-KCI-DEO-1-2S	NAL-AMP-FIS-STR-TET	feces/Chicken	SBA assay	[5]
6	*Gallinarum* (D)	287/91	Susceptible	Liver/Chicken	SBA assay	NCTC
7	*Gallinarum* (D)	A17-DW-005	STR-FIS-COL-NAL-CIP-GEN	Liver/Chicken	SBA assay	[29]

NA means not available; SBA means serum bactericidal activity assay; Nalidixic acid (NAL), neomycin (NEO), streptomycin (STR), tetracycline (TET), trimethoprim/sulfamethoxazole (SXT), ampicillin (AMP), sulfisoxazole (FIS), colistin (COL), ciprofloxacin (CIP), and gentamicin (GEN).

**Table 2:** Recovery of bacteria in the chickens’ post *S. typhimurium* challenge ^A^.

Group		Cloacal Swab				Liver	Cloacal Swab	Liver
		1 dpc	3 dpc	5 dpc	7 dpc			
1	PBS	6/6 ^B^	6/6	6/6	6/6	5/6 ^C^	2.50 ± 0.64 ^D^	1.46 ± 1.46
2	Killed ST	4/6	5/6	3/6	2/6	2/6	0.00 ± 0.00	0.00 ± 0.00
3	ST OMV	3/6	5/6	3/6	1/6	3/6	0.00 ± 0.00	0.00 ± 0.00

^A^ Challenge was performed with *S. typhimurium* 14028 strain using 1.0 × 10^9^ CFU at day 14 post-booster vaccination. ^B^ No. of positive samples after challenge strain recover. ^C^ No. of positive samples after enrichment culture. ^D^ Log10 (CFU/g).

**Table 3:** Recovery of bacteria in the chickens’ post *S. montevideo* and *S. albany* challenge ^A^.

Group		Cloacal Swab				Liver	Cloacal Swab	Liver
		1 dpc	3 dpc	5 dpc	7 dpc			
1	PBS + SM challenge	4/5 ^B^	4/5	3/5	1/5	2/5 ^C^	1.36 ± 1.17 ^D^	0.68 ± 0.83
2	ST-OMV + SM challenge	4/5	3/5	2/5	0/5	0/5	0.00 ± 0.00	0.00 ± 0.00
3	PBS + SA challenge	5/5	5/5	5/5	5/5	1/5	2.13 ± 0.50	0.34 ± 0.68
4	ST-OMV + SA challenge	4/5	4/5	3/5	2/5	0/5	1.20 ± 1.49	0.00 ± 0.00

^A^ Challenge was performed with *S. montevideo* A16-CF-111-L-1 and *S. albany* A16-CF-328-M-4 strains using 1.0 × 10^9^ CFU at day 14 post-booster vaccination. ^B^ No. of positive samples after challenge strain recover. ^C^ No. of positive samples after enrichment culture. ^D^ Log_10_ (CFU/g).

## Data Availability

Data are contained within the article.
